# The Nature of Knowledge and Epistemic Interests of Radiography Science—An Analysis of Doctoral Dissertations Using Critical Normative Epistemology Framework

**DOI:** 10.1111/scs.70105

**Published:** 2025-09-03

**Authors:** Törnroos Sanna, Leino‐Kilpi Helena, Siekkinen Mervi, Metsälä Eija

**Affiliations:** ^1^ Department of Nursing Science, Faculty of Medicine University of Turku Turku Finland; ^2^ School of Rehabilitation and Examination Metropolia University of Applied Sciences Helsinki Finland; ^3^ Turku University Hospital (TYKS) Turku Finland; ^4^ Western Finland Cancer Center Turku Finland

**Keywords:** dissertations, epistemic interests, epistemology, knowledge, radiography

## Abstract

**Background and Aim:**

Radiography science is a health sciences discipline and a knowledge system focusing on research into medical imaging and radiation therapy‐related phenomena: patient care, technology, safety and quality in these environments. This study aims to understand the nature of knowledge in radiography research by investigating epistemic interests and knowledge types.

**Methodological Design:**

The study used nursing science critical normative epistemology as a framework for the analysis. We used document analysis as a method, and the selected documents for the study were dissertations from the field of radiography between the years 1998 and 2020. The data corpus consisted of the methodology section of the dissertations and the study's aim and purpose. Data was analysed with an abductive analysis approach.

**Findings:**

The findings of the study indicate that radiography research has a dedicated type of knowledge according to epistemic interest. Knowledge is acquired through varying methodologies, and there does not seem to be any typical radiography methodology used, even though some methods are more common than others. According to our study, epistemic interests in radiography science are, in the majority of cases, inferential, that is, they aim to explain and explore phenomena within the domain of medical imaging and radiation therapy. However, there are also referential studies, aiming to understand different actors, processes and caring actions involved in the practice of this domain. Radiography research also furthers transformative interests, such as transformation focused on suppressed groups or practices in need of critical reflection. To a lesser extent, radiography research seems to further normative interests.

**Conclusions:**

Pragmatism describes the nature of radiography knowledge. The different knowledge types generated indicate a need for generalisable knowledge and subjective knowledge, as well as further critical reflection on the current practices in diagnostic imaging and radiotherapy.

## 1 Introduction

Radiography science is a health sciences discipline and a knowledge system in which the focus of the research is on medical imaging and radiation therapy related phenomena, such as patient care, technology, safety and quality in these environments [1, 2, 3]. Radiography is a human‐oriented discipline where creating a caring environment and caring actions, consisting of both technical competence and patient care, are essential [3, 4]. This article seeks to elucidate the nature of knowledge and epistemic interests of radiographic research. The study used Kim's nursing science critical normative epistemology [5] as a framework for the analysis. The framework integrates epistemological realism, emancipatory pragmatism and a normative perspective of human practice. Radiography science, similarly to nursing science, is pluralistic in its methodologies and human practice discipline [5, 6]. Therefore, this framework was found to be a suitable starting point for studying the nature of knowledge within radiography science, although some adjustments were necessary. The ontological commitments Kim [5] discusses were not particularly studied and an adjustment was made to one concept Kim uses in the framework. Kim [5] uses the concept of cognitive need when referring to different types of interests relating to knowledge types. As cognitive need is generally used when referring to individual perception, we decided to replace it with a concept of epistemic interest. Epistemic interest can be understood as something belonging to a knowledge system, such as an academic discipline [7].

Knowledge is sought by every academic discipline [8], and to qualify as scientific knowledge, knowledge must be gained through a systematic research process [9]. The concept of knowledge can be comprehended in three ways: personal knowledge, such as ‘I know Maria’, procedural knowledge, such as ‘I know how to ride a bike’ or propositional knowledge, such as ‘I know that X‐rays are a form of electromagnetic radiation’. The concept episteme refers to propositional knowledge, distinct from art or crafts and is therefore a suitable term when discussing knowledge produced by research [10].

The research process is directed by some epistemic interest with a set of objectives [7, 11]. Knowledge furthers a certain interest and is driven by a particular purpose. It can be said that any research involving humans is interest‐driven [11]. These interests should be transparently communicated [7].

Habermas [12] claimed that epistemic interests, or human knowledge interests, can be linked to three categories of research processes, each with specific logical‐methodological rules. These were the empirical‐analytical sciences with technical interest, historical‐hermeneutic sciences with practical interest, and critically orientated sciences with emancipatory interests.

To understand the nature of knowledge and epistemic interests, it is also necessary to understand the epistemological stances of research and the corresponding theories of explanation [13] (Table 1). Positivist epistemology has been predominant in empirical‐analytical sciences. Knowledge is generated through the creation of hypotheses, and then empirical observations verify or falsify this knowledge. The relationship between the knower and the known is objective and neutral. The ontological view in positivist epistemology is that of realism [16]. With historical‐hermeneutic sciences, interpretivist epistemology was introduced. Knowledge is generated by inductive reasoning, and it is subject to context, aiming to understand or discover new, unknown phenomena. The relationship between knower and known is subjective. The ontological view is relativist [17]. In critically oriented sciences, epistemology is guided by critical theory. The knowledge generated is influenced by issues of power, gender, culture or other social factors. The aim of knowledge is to change or to empower. The relationship between knower and known is subjective but also situational. The ontological view is that of historical realism [18].

**TABLE 1:** The connection between the different types of sciences, epistemology and mechanism of explanation [12, 13, 14, 15].

Type of science	Epistemology	Nature of explanation	Mechanism
Empirical‐analytical sciences	Positivist	Causal	Explains the phenomenon as an effect of a cause, in which the effect follows the cause. Is supported by probabilistic or statistical laws. Can be predictive
Historical‐hermeneutical sciences	Interpretivist	Phenomenological‐existentialist	Exploration and description of the phenomena within the context of the world of lived experience
Critically orientated sciences	Critical theory	Dialogical	Phenomena are explained as a chain of inferences, in dialogue with researchers and those being researched, considering the temporal and historical changes in processes

### 1.1 Epistemological Studies in Radiography

To understand what is already known about the epistemic interests of radiography science, a database search was made in Science Direct, PubMed and Scopus, using the search terms ‘epistemic OR epistemological OR epistemology OR knowledge’ AND interest AND radiography. Other than original research articles were excluded. Two relevant studies were found [2, 6]. An additional Google Scholar search revealed two other relevant studies [19, 20]. We also checked the references of these studies and one additional study was found [21].

The nature of academic radiographic knowledge has been described as extending over hard, applied to soft, pure categories, meaning it is situated somewhere between engineering and sociological sciences [21]. Studies into theoretical frameworks in these revealed that there is much imported knowledge and theoretical pluralism [19]. Radiographic research focuses on pragmatic aspects and applicable knowledge [2]. Lundgren and Lundén [20] studied radiography science epistemic concepts to reveal the substance of radiography from an ontological perspective.

According to Metsälä and Fridell [6], almost all knowledge interests in radiography research are either technical or practical, with only a minority presenting critical knowledge interests. The practical hermeneutic interests are connected to describing and understanding patient care‐related issues, whereas technical interests are to explain or develop medical, procedural, or technical solutions. Radiography seems to be almost equally aiming to investigate phenomena in caring as well as more technical phenomena [6]. Radiography seems to be flexible in the use of methodologies. Radiography seems to be pluralistic in its adoption of epistemologies, which seems to be in line with other health sciences [6, 10]. The study by Metsälä and Fridell [6] presented knowledge interests but not the type of knowledge with which these interests were associated. The nature of knowledge and epistemic interests needs further investigation.

Considering the importance of understanding the meaning of epistemology to a discipline of knowledge, very few studies have been conducted about the topic within radiography. A few reviews have been published, promoting qualitative epistemology within the dominant quantitative research tradition [22, 23, 24, 25]. Questions of how knowledge is in fact acquired, what kind of knowledge is produced or what purpose the research furthers within the discipline remain unanswered.

## 2 Aim and Research Questions

This study investigates the epistemic interests of radiography science, aiming to understand the nature of knowledge within the discipline.

The guiding research questions are as follows:

1. What epistemic interest does radiography research further?
2. With what kinds of knowledge types are these epistemic interests connected?

## 3 Methods

The methodology selected for the study was document analysis. Document analysis is a methodology by which new knowledge is generated by retrospectively examining documents produced for different purposes [26]. The documents analysed were dissertations from the field of radiography. We used a purposive sampling strategy for the selection of dissertations.

Dissertations were selected through Oadt and Proquest dissertation and thesis databases in May–July 2022. These databases were chosen because they are comprehensive resources for searching open‐access theses and dissertations worldwide. The dissertations were selected if their focus was on some area related to the core concepts of radiography identified and defined in a previous study [1]. The core concepts are the radiographers' profession, clinical practices in radiography, safe and high‐quality use of radiation and the technology used in radiography. The selected dissertations were from the fields of health or educational sciences. Medical or natural sciences (e.g., physics) dissertations were excluded, even if they studied topics related to the core concepts of radiography science; this was due to their perspective being different from the radiography science perspective. Altogether 53 dissertations were selected for analysis. The dissertations were published between the years 1998 and 2020. The data corpus in this study consists of the methodology section, the aim and purpose of the dissertations. The selected dissertations were published open access; for this reason, ethical approval was deemed unnecessary.

### 3.1 Theoretical Framework for Abductive Analysis

Data was analysed with an abductive analysis approach [27]. Abductive reasoning starts with a probable explanation but takes into account surprising findings that do not support the original theory [28]. Abductive analysis is a continuous iterative process, where data and theory interplay [27]. The framework of critical normative epistemology is based on four ontological commitments to knowledge content: human nature, human living, human practice and human agency. These ontological commitments are based on five types of epistemic interests: inferential epistemic interests, referential epistemic interests, transformative epistemic interests, normative epistemic interests and desiderative epistemic interests. According to Kim [5], these interests refer to the types of knowledge necessary for nursing practice. The types of knowledge are (1) generalised knowledge, (2) situated hermeneutic knowledge, (3) critical hermeneutic knowledge, (4) ethical knowledge and (5) aesthetic knowledge.

### 3.2 Abductive Analysis Process

After the first immersion with the documents, the data was analysed inductively without presumptions. Data was extracted into an extraction matrix created for this study. The extraction matrix contained columns for (1) aim and purpose, (2) goal of the study, (3) methodology used, (4) nature of reality (researchers' view), (5) sample, (6) research setting, (7) data collection methods, (8) data analysis methods, (9) nature of explanation and (10) epistemological stance. Analysis units were words or complete sentences answering each column of the extraction matrix. The trustworthiness of interpretations was secured by randomly selecting 10 dissertations. Two researchers (ST and EM) conducted the analysis blindly, and after which comparisons were made. There were differing views on a few interpretations, but these views were discussed, and agreement was reached. Researcher ST analysed the remainder of the documents. However, in uncertain or ambiguous situations, decisions were made within the entire research group.

**FIGURE 1:**
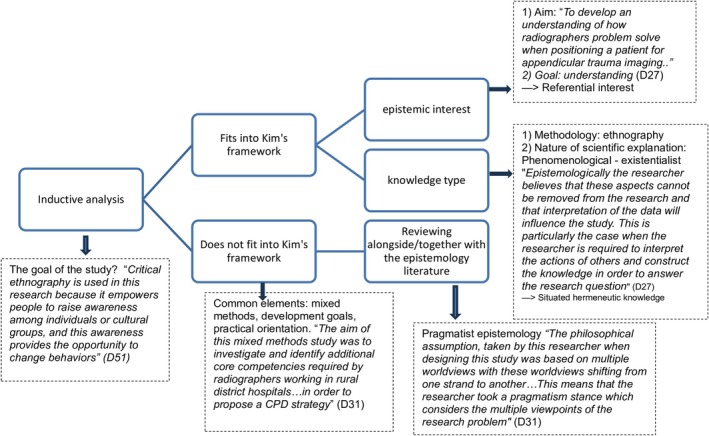
An example of an abductive analysis process.

In the second phase of the analysis, the data in the matrix were viewed in the light of Kim's (5) nursing epistemology framework. The data in the matrix were classified according to the five types of epistemic interests and associated knowledge types. We also searched for unexpected findings, in line with the abductive analysis process. Data that did not fit into Kim's theoretical framework was set aside at this point. These studies were analysed together by ST and EM, searching for similarities between the studies and together with theoretical epistemology literature (Figure 1).

The connecting threads to data unfit for Kim's theoretical framework seemed to be the mixed methods methodology used with the exploratory and developmental goal of radiography practice. This methodology was best fitted with the pragmatist epistemology framework. Pragmatism was first introduced by Pearce but later refined and interpreted by others, especially James and Dewey [29]. The basic tenet is those problems arise from action and the need to improve them. There might be a doubt about the practice; this doubt then launches an inquiry into the matter, aiming at a new, revised (better) practice. Interest arises from a specific problem, but there is no one way to solve the problem. Deductive, inductive and abductive inferences are equally important. The pragmatist way is to use methods that most likely solve the problem. The ontological position is realist, according to Pearce, but there are other pragmatists, such as James, who take a more relativist view [30].

A more modern pragmatist philosopher Van Fraassen [31] claims that an explanation is successful if it serves the cognitive and practical needs of scientists, such as making predictions, providing understanding, or facilitating control over a phenomenon. Researchers can choose which explanations to accept based on their pragmatic goals, even if those explanations do not necessarily reveal the ultimate truth about the underlying reality.

## 4 Findings

### 4.1 Overall View

The selected dissertations were from the United Kingdom (*n* = 31), the United States (*n* = 17), Australia (*n* = 2), Canada (*n* = 1), Hong Kong (*n* = 1) and South Africa (*n* = 1). Of the 53 dissertations (documents D1–D53, see S1), the goals of the selected studies were understanding (49%), exploration (19%), emancipation (15%), development (13%), explanation (11%) and prediction (6%). Some studies had more than one goal.

Methodologies varied, and some studies stated having used more than one methodology. The methodological approach was not always clearly stated. In these cases, we categorised them as either quantitative unspecified or qualitative unspecified. Altogether 22 dissertations used a quantitative methodology. There were 15 unspecified quantitative studies, three quasi‐experimental studies, a meta‐analysis, a cross‐sectional study, a controlled before‐and‐after study and a descriptive quantitative study. Altogether 34 dissertations used qualitative methodology, including unspecified qualitative studies (*n* = 6), case studies (*n* = 6), ethnographies (*n* = 4), grounded theory studies (*n* = 3), hermeneutic phenomenology studies (*n* = 3) and interpretative phenomenological analyses (*n* = 2). Single references were made to action research, symbolic interactionism, critical dramaturgy, historiography, constructivist grounded theory, descriptive phenomenology, an autoethnographic narrative inquiry, critical discourse analysis, and visual and critical ethnography methodologies. Five dissertations stated using a mixed methods methodology, three dissertations stated using a mixed methods case study and one stated that a multimethod methodology was used.

The ontological viewpoint was relativist in 24 dissertations (45%), realist in 18 (34%) and in 11 dissertations (21%), the viewpoint was not explicitly either.

In the majority of the dissertations, radiography professionals were the informants. Other informant groups were patients, students, educators, other healthcare staff, or managers. Some dissertations used only imaging phantoms without any human participants.

### 4.2 Epistemic Interests and Knowledge Types

The analysis of the aims, purposes and goals revealed the epistemic interests in the dissertations. Knowledge was acquired, for example, from patients undergoing radiographic procedures (D1), radiography processes (D2) and radiographers' perceptions (D10). Analysis of methodologies and the nature of scientific explanation in the dissertations revealed the knowledge types (Table 2).

**TABLE 2:** Radiography epistemic interests and knowledge types.

Realist ontology	Inferential epistemic interest: The goal of studies with inferential epistemic interests within radiography is to explain or explore phenomena in patterned ways. There is an attempt to predict how or why something happens/occurs. Examples of epistemic interests are: Explaining differences between groups (e.g., radiographer vs. radiologist reporting). Explaining the relationship between education and quality of care Prediction of radiographer behaviour Prediction of technology use. Exploring best practices Exploring risks related to radiography environment Exploring trends and relationships Optimisation of dose/protocol/process	Generalised knowledge: The nature of scientific explanation is logical‐empiricist. In prediction, causal inferences are drawn. Knowledge is developed using quantitative methodologies, from a positivistic epistemological stance. The knowledge produced provides generalisations, explanations of differences between groups or predictors of something.
Relativist ontology	Referential epistemic interest: The goal of studies with referential epistemic interests within radiography is to understand feelings, perceptions, thoughts or attitudes. The goal can also be to understand what influences something or to understand various expectations. There is an aim to explore phenomena with only scant prior theoretical knowledge. Examples of epistemic interests are: Exploring contemporary practice Understanding how actors themselves experience something Identifying or describing something about which little is known Conceptualising something	Situated hermeneutic knowledge: The nature of the scientific explanation is phenomenological‐existentialist. The knowledge produced focuses on subjective experiences in specific situations. Knowledge is developed using various qualitative methodologies, from an interpretivist epistemological stance. Understanding is possible through discovering similarities and differences through individual experiences.
Relativist ontology	Transformative epistemic interest: The goal of studies with transformative epistemic interests within radiography is to understand humans in social context, affected by issues of power, gender or structures. The goal can be the emancipation of professionals and patients alike. Examples of epistemic interests are: emancipating professional development Emancipating patient participation Correcting or understanding what influences distortions Understanding the phenomenon within its historical context of power relations	Critical hermeneutic knowledge: The nature of the scientific explanations is dialogical or interpretative. Knowledge is developed with qualitative methodologies, such as critical ethnography or discourse analysis, from critical or interpretivist epistemological stance. Knowledge is situated within its historical, cultural or social context.
Realist or relativist ontology	Normative epistemic interest: The goal of studies with normative epistemic interests within radiography is to understand or explore values, ethical behaviour or ethical practices. Examples of epistemic interests are: Exploring ethical behaviour of professionals Exploring professional values	Ethical knowledge: The nature of the scientific explanation can vary as ethical knowledge can be studied from varying/various methodological perspectives. The epistemological stance differs depending on the selected viewpoint.
	Desiderative epistemic interest: The goal of studies with desiderative epistemic interests within radiography is to understand practice, processes or culture of radiography in order to present an “ideal” or desiderative way of doing things. Examples of epistemic interests are: Cultural understanding Holistic developmental interest Conceptual understanding of idealistic model of practice	Aesthetic knowledge: The nature of the scientific explanation can vary but is inclined to the phenomenological‐existentialist view. The focus is on a holistic view of the processes: how care should be conducted and the cultural ethos of radiography. The epistemological stance is interpretative.
Realist ontology	Practical development interest The goal of studies with practical developmental interests is to explore practices and processes with an attempt to develop them. Examples of epistemic are: Implementing or establishing new protocol/scheme Developing practice/education/profession Designing new methods to measure or evaluate safety/quality	Applied knowledge This knowledge is developed mostly with mixed methods methodology, in line with the epistemological stance of pragmatism. Knowledge can Lead to a framework or model guiding the practice, or concrete products

*Inferential epistemic interests* were found in 16 dissertations. The goal of the six studies was to explain some radiographic phenomena. These included explaining differences between two groups of radiation therapists with different levels of education (D3), explaining whether radiographers and radiologists reporting differed (D5; D34), explaining usage and acceptance of technology (D14), professional knowledge updating (D18) and explaining the effect of education on the quality of care (D19). The goal of the three studies was to predict something. These included identifying the predictors related to knowledge, beliefs and the clinical settings for health promotion (D7), predicting the usage of technology to lower patient dosage (D22) and predicting safety deviance (D53). Seven studies had exploratory goals. These included exploring specialisation areas within magnetic resonance imaging (D12), processes of learning and development of problem‐solving skills (D15), simulation training for the development of critical thinking skills, self‐efficacy and competence (D21), interface pressure risk in medical imaging and radiotherapy environments (D33), inefficiencies in radiation therapy simulation (D36), the effect of positioning on the radiation dose and quality (D44) and exploring one technique over another when diagnosing (D45).

The knowledge produced in these studies was classified as *generalised knowledge*. Knowledge in all the studies had been gained by using quantitative, objective methodologies. Knowledge provided generalisations (e.g., D14), explanations of differences between groups (e.g., D19), or predictors of something (e.g., D22). The nature of the explanation was logical‐empiricist in all studies, in line with the positivistic epistemological stance.

*Referential epistemic interests* were found in 16 dissertations. The most common goal, altogether in 14 studies, was to understand some radiographic phenomena. Additionally, one study had an emancipatory goal together with the goal to understand the socialisation of radiographers (D38). Two studies were classified as having an exploratory goal: the exploration of contemporary radiography practice (D29) and the exploration of decision‐making processes (D39). There was a need to understand the effects of technology on practices (D4; D20), radiographers' perspectives on developing roles (D10; D23), how radiation therapists learn (D17), the expectations and experiences of radiographers (D25; D37), development of professionalism (D13), problem‐solving (D27), perceptions of professionalism (D47), coping and stress (D30) and an understanding of compassion and compassionate behaviours (D48; D49).

The knowledge produced in these studies was classified as s*ituated hermeneutic knowledge*. Knowledge had been gained through varying qualitative methodologies. Knowledge provided an understanding of the phenomena from a subjective or socially constructed view; for example, in dissertation D30, the lived experiences of radiation therapists were studied to better understand occupational stressors and coping mechanisms. The nature of the explanation in all studies was phenomenological‐existentialist and an interpretivist epistemological stance.

*Transformative epistemic interests* were found in nine dissertations. The goals of these studies were either to emancipate or to understand something from a historical, gendered, or structural position. There was a need to understand humanistic interaction with technology (D6), the development of specialisation from a gendered and hierarchical view (D9), interaction of radiation therapists and patients (D16), theory‐practice gap (D24), advanced roles (D35; D43) and how care is perceived and experienced (D42). There was a need to generate social change (D16), professional development (D35; D43), emancipate sexual minorities in professional groups (D41), understand care from the patient's point of view (D42) and empower cultural groups (D51).

These studies produced *critical hermeneutic knowledge*, that is, knowledge that had been gained from varying qualitative methodologies, with a critical epistemological stance. Knowledge is situated within its historical, cultural, or social context and cannot be understood without these structural elements being considered. For example, in dissertation D51, critical ethnography was used to give a voice to women of minority groups as to the reason why their mammography screening uptake is lower than women from higher socio‐economic groups. The nature of scientific explanation was dialectic in these studies.

There were only two dissertations with a *normative epistemic interest*. One study sought to understand how child protection is experienced among radiographers with the intention to affect attitudes (D28). The other study explored radiologic technologists' perceptions of professional values (D40). This type of knowledge can be classified as *ethical knowledge*. The nature of the scientific explanation was phenomenological‐existentialist in D28 and logical‐empiricist in D40. Epistemological stances were interpretivist and positivist respectively.

*Desiderative epistemic interests* were found in three dissertations. The goal of these studies was to understand radiographic examinations holistically (D1), to create a model of care in diagnostic radiography (D2) and to understand cultural silences (D46). These interests were seen as being associated with *aesthetic knowledge*. The nature of the scientific explanation was phenomenological‐existentialist in D2 and D46, and more inclined to logical‐empiricist in D1.

The epistemic interests in seven dissertations did not directly fit into the five types of interests (5) but rather contained elements of two or more different types of interests. Common to all was that they had a *practice‐orientated developmental interest*. These included exploring the opinions of relevant stakeholders for developing a continuous professional development (CPD) model in radiography (D8), exploring the impact of technology to develop radiography education and practice (D11), developing a new product to measure radiation in tissue (D26), developing a quality framework (D32), exploring the landscape of rural radiographers in order to develop a CPD model (D31), developing a new method to test safety and quality in radiography environments (D50) and improving emerging practices (D52). Knowledge produced in these studies was classified as *applied knowledge*. The nature of explanation varied, and the epistemological stance was pragmatist.

## 5 Discussion

This study is the first, as far as we know, that demonstrates the different types of knowledge produced by research into radiography science. The study confirms radiography science as a discipline with pluralist epistemologies, in line with previous studies [6, 19]. It also confirms Castle's claim [21] that the nature of radiographic knowledge is situated somewhere between applied hard and soft pure sciences, and does not fit rigidly into either group.

There seems to be a need to produce different types of knowledge within radiography research to accommodate the different epistemic interests. Generalisable knowledge is needed to explain phenomena within the radiography domain and to make predictions. Caring phenomena, perceptions, feelings and attitudes in diagnostic imaging or radiation therapy are best understood through situated hermeneutic knowledge. Critical hermeneutic knowledge can fulfil the transformative interests when changes to power structures or empowerment needs must be addressed. Applied knowledge is of use to application in the direct practical developmental interests of radiography.

There are different paths to knowledge within radiography. Knowledge in radiography science is acquired through various methodologies and no typical radiography methodology seems to be used, even though some methods are more common than others. In this study, unspecified quantitative studies using cross‐sectional surveys were the most frequent. In the qualitative methodologies, case studies were the most commonly used. This is a sign of a young discipline, where theoretical knowledge is scant and there are multiple unanswered problems [32]. It might also be an indication of radiography science adopting pragmatist epistemology, where the nature of the problems to be solved defines the methodological choices rather than establishes the practices [31], and the problems mainly emanate from the practices [30]. In addition, complex problems, as those faced within the health care environment, require synthesised health sciences knowledge, emanating from both natural and social science traditions [3, 33].

In fact, pragmatist epistemology seems to fit well with radiography research, as it does in other human practice disciplines, such as nursing [34] and social work [35]. Kim [5] also adopted elements of pragmatism and normative ideals in her framework for nursing epistemology; this framework was used as the starting point of the analysis made in this study. According to our study, epistemic interests in radiography are equally inferential and referential. Inferential aims to explain and explore phenomena within the domain of medical imaging and radiation therapy, and referential aims to understand the different actors, processes and caring actions in the practice of this domain. Radiography research also addresses transformative interests, that is, transformation focused on suppressed groups or practices in need of critical reflection. Practical development interests explore practices and processes with an attempt to generate development. To a lesser extent, radiography research seems to address normative interests. Therefore, it can be said that pragmatism describes the nature of radiography knowledge better than normative.

Radiography practice seems to be very similar all around the globe despite educational differences, different historical starting points and the different names given to essentially the same discipline [36]. If the practice is similar, then as a knowledge system, radiography research should be serving the same epistemic interests. Locally, of course, some problems need more solving than others. As Wilholt [11] expressed, the epistemic interests need to be transparently communicated in order to secure the trustworthiness of scientific research. Another reason to communicate these interests is the accountability required in the name of sustainable science [37]. Critical epistemological discussions are needed in order for radiography as a discipline to evolve into a coherent knowledge system.

## 6 Study Limitations

The data, obtained from the 53 dissertations, presents a small sample of all the research done within radiography. Every year, a considerable number of studies are published in radiography and other relevant peer‐reviewed journals. However, similar results of methodological and theoretical pluralism [6, 19] confirm that even with a larger sample, the results might not differ very much. It is also noteworthy that in doctoral dissertations, the development of the discipline is more clearly visible, as the theses are a demonstration by doctoral candidates of their ability to complete complex research and to contribute to the field of knowledge in this particular discipline [38, 39].

The epistemological and ontological stances of the dissertations were not always coherent with the study aims and methods used; this required decisions to be made on how to classify the studies based on the overall construct of the thesis. The authors of the dissertations might, in some cases, disagree with the choices made in this study.

## 7 Conclusion

The nature of radiography knowledge fits into a critical normative epistemology framework but is more pragmatically orientated. There is no one scientific explanation model that reveals the truth about the underlying reality. Reality can be understood as realistic or relativist, depending on the phenomena to be studied. The different knowledge types generated within radiography science indicate there is a need for generalisable knowledge and more subjective knowledge, as well as further critical reflection on the current practices in diagnostic imaging and radiotherapy. Essentially, radiography is a discipline where new knowledge is generated to better diagnostics, health and care of patients.

## Author Contributions

Sanna Törnroos: research design, data acquisition, analysis of data, drafting the work. Helena Leino‐Kilpi: research design, critical review. Mervi Siekkinen: research design, critical review. Eija Metsälä: research design, analysis of data, drafting the work. All authors have contributed to the design of the work and have revised the manuscript and added to the intellectual content of the work. All authors have approved the version of the manuscript to be submitted and are accountable for the accuracy and integrity of the manuscript.

## Disclosure

The authors declare that we did not use any generative Artificial Intelligence (such as ChatGPT) in any stage. NVivo was used to manage documents in the analysis phase, but it was not used to assist the analysis in any other way.

## Ethics Statement

This study did not require ethical board approval. This study did not include human or animal participants. The study was based on publicly open documents. We authors consciously assure that for the manuscript ‘The Nature of Knowledge and Epistemic Interests of Radiography Science—An Analysis of Doctoral Dissertations Using Critical Normative Epistemology Framework’ the following is fulfilled:

(1) This material is the authors' own original work, which has not been previously published elsewhere.

(2) The paper is not currently being considered for publication elsewhere.

(3) The paper reflects the authors' own research and analysis in a truthful and complete manner.

(4) The paper properly credits the meaningful contributions of co‐authors and co‐researchers.

(5) The results are appropriately placed in the context of prior and existing research.

(6) All sources used are properly disclosed (correct citation). Literally copying of text is indicated as such by using quotation marks and giving proper reference.

(7) All authors have been personally and actively involved in substantial work leading to the paper, and will take public responsibility for its content.

## Conflicts of Interest

The authors declare no conflicts of interest.

## Supporting information


**Data S1:** Supporting Information.

## Data Availability

The data that supports the findings of this study are available in the Supporting Information of this article.
